# GlucoMedix®, an extract of *Stevia rebaudiana* and *Uncaria tomentosa*, reduces hyperglycemia, hyperlipidemia, and hypertension in rat models without toxicity: a treatment for metabolic syndrome

**DOI:** 10.1186/s12906-022-03538-9

**Published:** 2022-03-08

**Authors:** León F. Villegas Vílchez, Julio Hidalgo Ascencios, Thomas P. Dooley

**Affiliations:** 1 Department of Cellular and Molecular Sciences, Section of Pharmaceutical Sciences, Faculty of Sciences and Philosophy, Universidad Peruana Cayetano Heredia, Lima, Peru; 2 Quality Control Service, Research and Development Laboratories, Universidad Peruana Cayetano Heredia, Lima, Peru; 3 Research and Development, LivFul Inc, Cheshire, UK

**Keywords:** Diabetes, Hyperlipidemia, Hypertension, Metabolic syndrome, Oxindole alkaloid, Steviol glycoside, *Stevia*, *Uncaria*, Cat’s claw

## Abstract

**Background:**

The objective of this in vivo study is to evaluate in five rat models the pharmacologic effects and toxicity of a commercial hydro-alcoholic extract, GlucoMedix®, derived from *Stevia rebaudiana* and the pentacyclic chemotype of *Uncaria Tomentosa* (Willd.) DC, for use as a treatment for metabolic syndrome. The extract contains phytochemicals of *Stevia* (e.g., steviol glycosides) and *Uncaria* (e.g., pentacyclic oxindole alkaloids, but lacks tetracyclic oxindole alkaloids).

**Methods:**

The pharmacologic assessments in three rat models include reductions in chemically induced hyperglycemia, hyperlipidemia (cholesterol and triglycerides), and hypertension, all of which are comorbidities of metabolic syndrome. Acute toxicity and 28-day subacute toxicity were assessed in rat models at doses higher than those used in the efficacy models.

**Results:**

The acute oral toxicity was evaluated in Holtzman rats and the extract did not produce acute toxic effects or lethality, with the LD_50_ > 5000 mg/kg (extract wet weight). Furthermore, subacute oral toxicity was evaluated in rats for 28 days at daily doses as high as 2000 mg/kg without toxicity or abnormal clinical chemistry or hematological effects. Daily oral doses of 250 - 1000 mg/kg were used to evaluate the treatment effects in hyperglycemic (alloxan-induced and glibenclamide-controlled), hyperlipidemic (cholesterol-induced and atorvastatin-controlled), and hypertensive (L-NAME-induced and enalapril-controlled) rat models. Alloxan-induced hyperglycemia was reduced in a dose-dependent manner within 28 days or less. Cholesterol-induced hyperlipidemic rats exhibited dose-dependent reductions in cholesterol and triglycerides at 21 days. Furthermore, GlucoMedix® produced a dose-dependent decrease in systolic and diastolic arterial blood pressure in L-NAME-induced hypertensive rats at 28 days.

**Conclusions:**

The five in vivo rat models revealed that the all-natural phytotherapy GlucoMedix® is a safe and effective treatment for hyperglycemia, hyperlipidemia, and hypertension. This extract is expected to affect multiple comorbidities of metabolic syndrome, without any acute or subacute oral toxicity in humans. Although multiple prescription drugs are well known for the treatment of individual comorbidities of metabolic syndrome, no drug monotherapy concurrently treats all three comorbidities.

## Background

Metabolic syndrome is a cluster of comorbidities that leads to an increased risk of cardiovascular, cerebrovascular, and metabolic diseases [1]. These factors include abdominal obesity, hyperlipidemia, type 2 diabetes, and hypertension. Metabolic syndrome is common and affects 34% of adults in the USA [2] and 27% in Peru [3]. Hyperlipidemias are characterized by elevated total cholesterol, triglycerides, very low-density lipoprotein cholesterol (VLDL), low-density lipoprotein (LDL) cholesterol, free fatty acids, and apolipoprotein B levels, as well as reduced levels of high-density lipoprotein (HDL) cholesterol. Type 2 diabetes is characterized by chronic hyperglycemia resulting from defects in insulin action upon target cells. Hypertension manifests as elevated systolic and diastolic arterial blood pressure.

Pharmacological interventions, as well as diet and exercise, can treat these three conditions. However, no single drug has been identified that effectively treats all three indications. Given (a) the unmet medical need for effective and safe treatments for metabolic syndrome, and (b) that selected drugs can exhibit side effects, many individuals are more interested in the use of traditional medicinal plants or herbal extracts.

*Stevia rebaudiana*, a sweet herb native to South America, has long been used by the indigenous peoples for a variety of medical conditions, including diabetes and hypertension. Stevioside and rebaudioside A are the two most abundant steviol glycosides present in dried *Stevia* leaves and are responsible for their intensely sweet taste. In addition, various pharmacological effects of *Stevia* and steviol glycosides have been identified in animal models and humans, such as antibacterial, anti-caries, anti-edema, antifungal, and antihypertensive effects [4–10], anti-hyperlipidemia effects [11, 12], and anti-hyperglycemic effects [6, 11, 13–18]. In view of its anti-hyperglycemic and antihypertensive effects, *Stevia* has been suggested as a possible treatment for metabolic syndrome [19, 20].

*Uncaria tomentosa* (cat’s claw) is commonly used to treat various diseases by some South American indigenous people groups such as arthritis [21], heart disease, tumors [22], inflammatory conditions [23], among other conditions [24, 25]. *Uncaria* has antioxidant [26], antiviral [27], and immunomodulatory properties [28]. Of relevance to the current study, *Uncaria* has demonstrated anti-hyperglycemic activity [29–33].

The medicinal use of a plant or its extracts depends on the different chemical compounds present such as oxindole alkaloids, terpenic glycosides, sterols, flavonoids, flavons, and phenols. Oxindole alkaloids are classified into two major chemotypes: tetracyclic oxindole alkaloids (TOA) and pentacyclic oxindole alkaloids (POA). TOAs act primarily on the central nervous system, while the POAs affect the cellular immune system [34–36]. The interaction of tetra- and pentacyclic alkaloids can be antagonistic. Therefore, the determination of the content of the TOAs and POAs (e.g., within *Uncaria* extracts) is essential for studies of pharmacologic and toxicologic properties. Also, oxindole alkaloids in wild populations of *Uncaria tomentosa* in South America are variable [37].

A hydro-alcoholic extract of *Stevia rebaudiana* and *Uncaria tomentosa*, GlucoMedix®, was developed to address hyperglycemia and metabolic syndrome. This research project aimed to evaluate in five rat animal models the acute and subacute oral toxic effects, as well as the anti-hyperglycemic (alloxan-induced and glibenclamide-controlled), antihypertensive (L-NAME-induced and enalapril-controlled), and anti-hyperlipidemic (cholesterol-induced and atorvastatin-controlled) activities. The overall goal was to demonstrate the safe and effective use of GlucoMedix® in the aggregate rat models of toxicology and efficacy, thus providing a clinical bridge rationale for the treatment of metabolic syndrome in humans and with a suggested allometrically-scaled starting dose.

## Methods

### Materials

Organic solvents were purchased from Merck (USA). Isopteropodine (Uncarine E) and cat’s claw powder standards were purchased from the United States Pharmacopeia (USP, USA). GlucoMedix® hydro-alcoholic extract (23% ethanol in mineral water) of *Uncaria Tomentosa* (Willd.) DC (pentacyclic chemotype; Samento® brand) and *Stevia rebaudiana* was obtained from NutraMedix Inc. (Jupiter, FL, USA).

### Phytochemicals within GlucoMedix®

GlucoMedix® contains 15% v/v of *Uncaria* bark extract and 11.67% w/v of *Stevia* leaf extract powder. The content of steviol glycosides is 8.18% w/v (analysis by KML Laboratories Inc., Bonners Ferry, Idaho).

To demonstrate that GlucoMedix® contained known phytochemicals from both *Uncaria* and *Stevia* a qualitative HPLC-MS-MS analysis was performed. 100 ul of homogenized GlucoMedix® was diluted 1:20 in methanol:water (1:1), filtered with a 0.25 um filter. Ultra-high performance liquid chromatography was performed with a Dionex Ultimate 3000 UHPLC (Thermo Scientific); injection volume of 4 ul; Luna Omega C18 100 Angstrom Phenomenex column at 40C; eluent A – water:1% HCOOH and eluent B – acetonitrile:1% HCOOH. The mass spectrometer was a Q Extractive Plus (Thermo Scientific) and using ESI positive and negative modes.

To demonstrate that GlucoMedix® contained pentacyclic oxindole alkaloids and lacked tetracyclic oxindole alkaloids, HPLC analyses were performed with a 1200 Series HPLC (Agilent Technologies, USA) equipped with a degasser, quaternary pump, automatic sampler, column oven, and photodiode array detector (DAD). The analyses were carried out using the USP 42 method. In summary, a C18 column was used (150 mm × 4.6 mm, 3 μm). The mobile phase consisted of 10 mM phosphate buffer, pH 7.0 (A), acetonitrile (B), and methanol and glacial acetic acid (99: 1) (C). Gradient composition (A: B: C): 0-17 min (65:35:0); 17–25 min (50:50:0); 25-30 min (50:50:0); 30–31 min (0:0:100); 31–36 min (0:0:100); 36–39 min (65:35:0); 39–49 min (65:35:0). The flow rate was 0.75 ml/min, detection was made at 245 nm with a constant temperature of 25 °C and the injection volume was 10 uL. The total contents of pentacyclic oxindole alkaloids (POA) and tetracyclic oxindole alkaloids (TOA) were calculated by the sum of the contents of individual alkaloids, namely speciesophylline, uncarine F, mitraphylline, isomitraphylline, pteropodine, and isopteropodine for POAs, and rhyncophylline and isorhyncophylline for TOAs. The results were expressed as mg/100 ml of the mean value of three determinations using isopteropodine (USP, USA) as the external standard.

### Laboratory animals

Male and female Holtzman albino rats of 8-10 weeks of age weighing 220-260 g were used. The rats came from the laboratory animal facilities of the Research and Development Laboratories of the School of Science and Philosophy of the Universidad Peruana Cayetano Heredia. The rats were kept under an automatic cycle of light and dark (12:12), temperature (23 ± 2 °C), and a relative humidity less than 70%. The animals were fed a basic rodent diet and water ad libitum, with a 1-week acclimatization period. The animals were fasted for 12 h before the experiment(s). The experimental protocols (SIDISI no. 207690) were approved by the Ethics Committee for Animal Experimentation of the Universidad Peruana Cayetano Heredia.

### Acute oral toxicity

The acute oral toxicity study of GlucoMedix® was evaluated according to OECD guideline 423 – “Acute Oral Toxicity” [38] on male Holtzman rats, where the maximum limit test dose of 5000 mg/kg was used. All the animals were kept overnight fasting before every experiment, with free access to water. The animals were divided into four groups, each comprising 3 animals. The 1st group served as a negative control, while 2nd, 3rd and 4th were treatment groups received orally GlucoMedix® (diluted in distilled water) at dose of 2000 mg/kg, another dose of 2000 mg/kg, and 5000 mg/kg. The OECD guide mentions that two doses are required, thus the 2000 mg/kg dose was tested twice. The highest dose of 5000 mg/kg was selected according to the guide’s annex. Lower doses were not necessary as per the recommendations of the animal welfare principles of the 3Rs - replacement, reduction, and refinement.

Before dose administration, the body weight of each animal in grams (to two decimals) was determined, and the dose was calculated according to the body weight. The animals were observed for any toxic effect for the first 4 h after the treatment period. Furthermore, animals were investigated for a period of 14 days for any toxic effect. Behavioral changes and other parameters were assessed, such as body weight, urinations, food intake, water intake, respiration, convulsion, tremor, temperature, constipations, changes in eye and skin colors, death, among others.

### 28-day subacute oral toxicity study

The study was performed according to the OECD Guideline 407 - “Repeated Dose 28-day Oral Toxicity Studies of Drugs” [39]. Eight-week-old Holtzman albino rats were housed in the same conditions as described above. The 40 animals were randomly divided into four groups containing 10 rats each (5 females and 5 males). GlucoMedix® diluted in distilled water was administered to groups of rats at the concentrations of 250, 1000, and 2000 mg/kg by gavage of 10 mL/kg for 28 days. The control group received the vehicle only. The animals were observed for signs of toxicity and mortality throughout the experimental period. The weight of each rat in grams (to two decimals) was recorded at weekly intervals throughout the course of the study. At the end of the 4-week experiment, the animals, fasted for 12 h, obtaining blood samples from treated animals, first by blood extraction from the retro-orbital plexus and then by cardiac puncture. Blood was collected into capillaries for microhematocrit, for the determination of the hematological examination and tubes containing EDTA were processed immediately for biochemical analysis (“clinical chemistry”). The tubes were centrifuged at 3000×g at 4 °C for 10 min to obtain plasma (stored at − 20 °C until analysis).

Hematological analysis was performed using an automatic hematological analyzer. Parameters included red blood cell (RBC) count, white blood cell (WBC) count, WBC differential counting [neutrophils (NEU), lymphocytes (LYM), monocytes (MONO), eosinophils (EOS)], hemoglobin (HGB), and hematocrit (HCT). For biochemical analysis (“clinical chemistry”) the following parameters were determined: glucose (GLU), blood urea nitrogen (BUN), creatinine (Crea), aspartate aminotransferase (AST), alanine aminotransferase (ALT), total cholesterol (T-Chol), triglycerides (TG), and thyroid hormones T3, T4 and thyroid stimulating hormone (TSH). These levels were determined using an autoanalyzer.

All animals were subjected to necropsy at the end of the toxicity studies. Necropsy was performed to analyze the macroscopic external features of the lungs, heart, stomach, intestine, testes, liver, kidneys, and bladder. These organs were carefully removed and the tissue samples were fixed in 10% formalin, sectioned, and stained with hematoxylin and eosin (H & E) for microscopic histopathological examination.

### Anti-hyperglycemic activity

The Holtzman rats were induced to be hyperglycemic by intraperitoneal administration with a dose of alloxan (150 mg/kg) dissolved in saline solution. Alloxan destroys pancreatic beta cells, the source of insulin, thus mirroring a type 1 diabetic condition. It should be noted however that some researchers assert it is a type 2 diabetes model [14, 17]. After 7 days, the animals were fasted for sample taking. To be considered chemically positive for hyperglycemia, the blood glucose level must be greater than 200 mg/dL, whereas < 70 mg/dL denotes hypoglycemia.

Forty-two male Holtzman rats were used and kept in individual cages in groups of seven animals each. Induced (hyperglycemic) rats were used, which were administered 1 mL of the sample orally (diluted in distilled water), for 28 days at the doses of 250, 500, and 1000 mg/kg body weight, a control group given distilled water, and a positive control group treated with glibenclamide (10 mg/kg). Glibenclamide (Glyburide) is a sulfonyl urea that stimulates insulin production, and in this model via the remaining pancreatic beta cells post-alloxan treatment. The blood samples were taken from the retro-orbital sinus of the eye of the rats. Blood glucose values were measured in mg/dl (with no decimal) at 0, 7, 14, 21, and 28 days.

### Anti-hyperlipidemic activity

Forty-two male Holtzman rats were used in six groups of seven rats per group. One group served as the normal (baseline) control and did not receive the induction agent. Five groups received cholesterol at a dose of 80 mg/kg, diluted in gum tragacanth 2% (emulsifier), by oral administration to induce hyperlipidemia.

The animals were fasted overnight before blood collections. After successful induction of hypercholesterolemia, when blood levels of cholesterol and triglycerides increase to > 200 mg/dl, daily oral treatment with GlucoMedix® was administered for up to 21 days. Of the five groups that were induced, one group received only normal saline solution and was considered as the induced control group; it served as the negative control. Another group received atorvastatin, a cholesterol-reducing statin drug, at a daily oral dose of 20 mg/kg via the oral route; it served as the positive control. The remaining three groups received GlucoMedix® at a daily oral dose of 250, 500, and 1000 mg/kg, via the oral route. All groups had free access to water and food during the study period.

Blood samples were taken from the retro-orbital sinus of the eye of the rats before the induction of hyperlipidemia (basal), after the induction of hyperlipidemia, and at day 21 of the oral treatments. Blood samples were centrifuged to obtain the serum. Sera were subjected to a biochemical test using Valtek Diagnostics reagents for total cholesterol (CHOD-PAP method) and triglycerides (GPO-PAP method), readings were taken at 505 and 520 nm on a Hewlett Packard spectrophotometer model HP8453 (USA). Values were measured in mg/dl (to one decimal).

### Antihypertensive activity

The hypertension model using L-NAME (non-selective nitric oxide synthase inhibitor) is widely used to study the pathophysiology and pharmacology of high blood pressure. The administration of L-NAME produces a 20 to 40% increase in systolic and diastolic blood pressure in rats. In addition, L-NAME produces cardiac fibrosis and nephropathy, target organ damage characteristics that are similar to human hypertension. L-NAME hypertension is mainly due to vasoconstriction, as it decreases NO synthesis and increases renin synthesis. Because of these mechanisms, the administration of an angiotensin converting enzyme (ACE) inhibitor together with L-NAME prevents the development of high blood pressure and target organ damage in this model. Enalapril, an ACE inhibitor, serves as the positive control to reduce arterial blood pressure in this model. Forty-two male Holtzman rats were randomized into groups for daily administration of the following treatments:

- Group 1. Saline solution 0.9% (1 ml i.p.) + Saline solution 0.9% (1 ml p.o.); uninduced.
- Group 2. L-NAME (40 mg/kg i.p.) + Saline solution 0.9% (1 mL p.o.); induced, negative control.
- Group 3. L-NAME (40 mg/kg i.p.) + Enalapril (25 mg/kg p.o.); induced, positive control.
- Group 4. L-NAME (40 mg/kg i.p.) + GlucoMedix® (250 mg/kg p.o.); induced, treated.
- Group 5. L-NAME (40 mg/kg i.p.) + GlucoMedix® (500 mg/kg p.o.); induced, treated.
- Group 6. L-NAME (40 mg/kg i.p.) + GlucoMedix® (1000 mg/kg p.o.); induced, treated.

A Panlab Harvard Apparatus (www.Seca.Com software) blood pressure measuring equipment was used, which contains a microprocessor or sensor to indirectly capture blood pressure values in the rat tail, recording systolic and diastolic blood pressure values expressed in mmHg (with no decimal). The rats were placed in a trap that immobilizes them during the test. The animals were acclimated to the restraint box for 30 min, three days prior to the test. On the day of the test, they were kept there again and after the administration of the sample in its different concentrations, five measurements were taken and the average of five consecutive readings was calculated. Values on days 7, 14, 21, and 28 of treatment initiation were used to evaluate the antihypertensive effect.

### Statistical analysis

The results obtained in the rat animal models were statistically analyzed to evaluate the effect of the experimental treatments and controls (negative and positive), with significance levels of 95% (*p* < 0.05 or < 0.001), using ANOVA and a multiple comparison test (Tukey’s test), to determine if significant differences exist between the groups. The Anderson Darling Test was applied to establish a normal distribution.

## Results

### Phytochemicals within GlucoMedix®

**Fig. 1:**
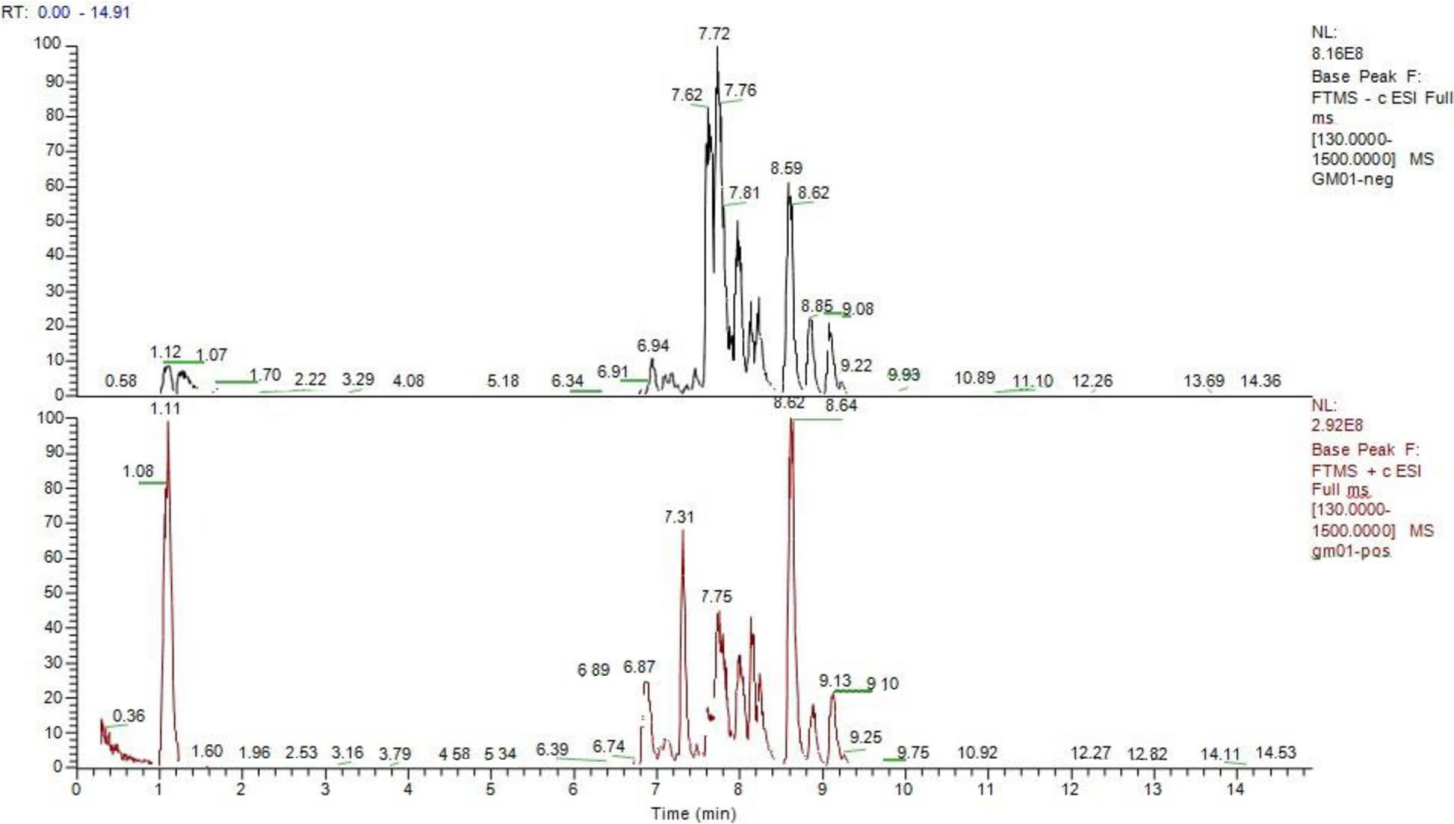
HPLC-MS total ion current chromatograms of phytochemicals within GlucoMedix® in ESI positive mode (top) and ESI negative mode (bottom)

To demonstrate qualitatively that known phytochemicals from both *Uncaria* and *Stevia* were present within the combination extract an analysis by HPLC-MS-MS of GlucoMedix® has been performed. From the chromatograms in Fig. 1, thirteen components of the multiple peaks have been positively identified using this method. Among these, four phytochemicals are known to be from *Uncaria*: Uncarine C, Isomitraphylline N-oxide, Uncarine D, and 3-Hydroxy-12-ursene-27, 28-dioic acid 3-O- [Glucopyranosyl- (1 → 3) -fucopyranoside], 28-O-glucopyranosyl ester. Nine phytochemicals are known to be from *Stevia*: Rebaudioside D, Rebaudioside A, Stevioside, Rebaudioside C, Dulcoside A, Stevioside isomer, Rubusoside, Rebaudioside B and Steviobioside. Relative quantities among the 13 identified chemicals were not determined, as the intensity of the peaks in the chromatograms can be dependent upon the structural characteristics of the chemicals. However, outside of the HPLC-MS approach, in relative terms the *Stevia*-derived phytochemicals in aggregate are more abundant, based upon the overall composition (i.e., 11.67% w/v of *Stevia* leaf extract powder, resulting in a steviol glycosides content of 8.18% w/v).

**Fig. 2:**
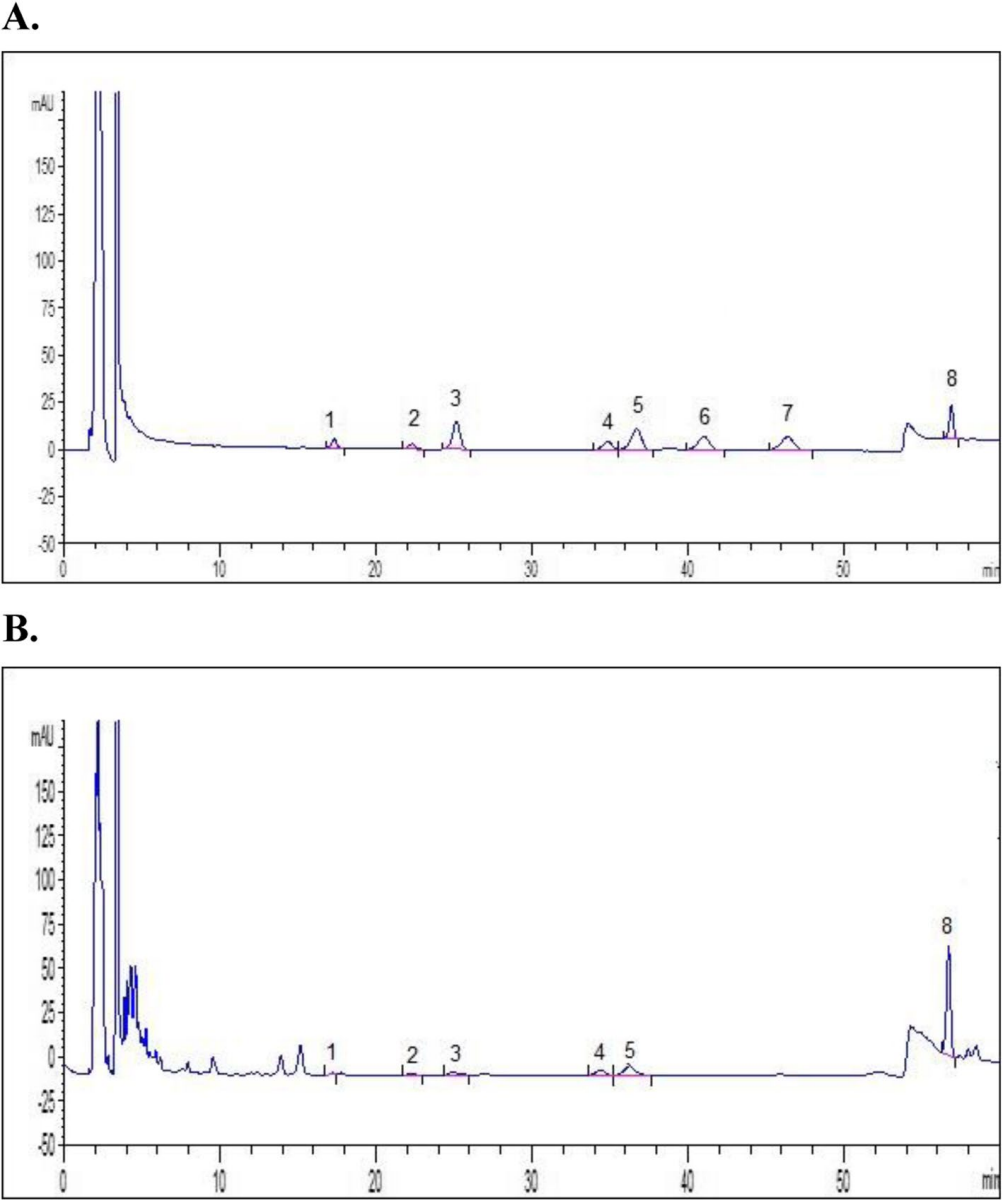
HPLC chromatograms of oxindole alkaloids within GlucoMedix®. HPLC chromatograms of (**A**) USP standard of *Uncaria tomentosa* bark and (**B**) GlucoMedix®: Peaks 1. Speciophylline, 2. Uncarine F, 3. Mitraphylline, 4. Isomytraphylline, 5. Pteropodine, 6. Rhynchophylline, 7. Isorhynchophylline, and 8. Isopteropodine

To further characterize the oxindole alkaloids, HPLC analysis of GlucoMedix® (Fig. 2) revealed a content of 2.56 ± 0.080 mg/100 mL for pentacyclic oxindole alkaloids (POAs) and the absence of tetracyclic oxindole alkaloids (TOAs; rynchophylline and isorhyncophylline). The results indicate that the individual content (mg/100 ml) of POAs are speciophylline 0.035 ± 0.011, uncarine F 0.051 ± 0.012, mitraphylline 0.125 ± 0.013, isomitraphylline 0.184 ± 0.016, pteropodine 0.471 ± 0.039 and isopteropodine 1.698 ± 0.051. Therefore, the predominant oxindole alkaloids in the GlucoMedix® extract are isopteropodine and pteropodine. Between the two methods (HPLC and HPLC-MS-MS) the results affirm the inclusion of the pentacyclic chemotype of cat’s claw, and a total of 9 distinct *Uncaria* chemicals have been identified.

### Acute oral toxicity

The rat animal model did not show any signs of acute toxicity after the oral gavage treatment with GlucoMedix®. The LD_50_ obtained was higher than 5000 mg/kg. In fact, no animals died or manifested illness at this highest dose. Well-being parameters such as sleep, behavioral pattern, motor activity, skin, coat, and appetite used to assess toxicity were found to be normal up to a dose level of 5000 mg/kg during the observation time of the test.

There was no weight loss observed in the treated and control animals at one and two weeks of observation (Table 1). All treated groups of animals continued to gain weight at the same rate as the control group. The distribution of all the means of the body weights of the animals for each concentration has been made by the Anderson Darling Test. The distribution of the means of the weights is normal. So, there are no statistically significant differences between the group averages.

**Table 1:** Body weights (g) of rats before and after acute oral treatment with GlucoMedix®

Groups	0 days	7 days	14 days
**Control**	210.61 ± 1.17	214.46 ± 2.59	218.88 ± 1.96
**2000 mg/kg**	210.54 ± 1.87	214.50 ± 0.98	219.24 ± 0.68
**2000 mg/kg**	211.85 ± 1.08	215.68 ± 1.57	219.86 ± 1.48
**5000 mg/kg**	212.80 ± 0.98	217.16 ± 2.04	220.97 ± 2.26

Values are expressed as mean ± SD. *n* = 3. There is no significant difference between the body weights of the control and treated groups at 14 days

The maximum dose tested is 5x higher than the maximum dose used in the three rat efficacy models (below) and is 2.5x higher than the maximum dose used in the 28-day subacute toxicity study (below).

### 28-day subacute oral toxicity

During 28 days of administration of GlucoMedix® no death or adverse clinical signs were observed in rats who received 250, 1000, and 2000 mg/kg in both sexes. There was no significant change in body weight in either sex compared with the untreated control group (Tables 2 and 3). All treated groups of animals continued to gain weight at the same rate as the control group over four weeks. The distribution of all the means of the body weights of the animals for each concentration has been made by the Anderson Darling Test. The distribution of the means of the weights is normal. So, there are no statistically significant differences between the group averages.

**Table 2:** Body weights (g) of male rats before and during 28-day sub-acute oral treatment with GlucoMedix®

Groups	0 days	7 days	14 days	21 days	28 days
**Control**	212.34 ± 0.83	216.51 ± 0.69	222.42 ± 1.39	231.84 ± 1.54	238.73 ± 0.80
**250 mg/kg**	213.30 ± 2.76	216.49 ± 2.63	221.59 ± 2.38	229.38 ± 2.72	238.10 ± 1.40
**1000 mg/kg**	212.20 ± 1.79	216.7 ± 1.17	221.82 ± 1.11	230.68 ± 1.60	238.83 ± 1.88
**2000 mg/kg**	212.45 ± 1.98	217.23 ± 1.60	222.28 ± 1.76	229.85 ± 1.10	237.54 ± 1.11

Values are expressed as mean ± SD. *n* = 5. No significant difference between the weights compared to the control group at 28 days

**Table 3:** Body weights (g) of female rats before and during 28-day sub-acute oral treatment with GlucoMedix®

Groups	0 days	7 days	14 days	21 days	28 days
**Control**	204.19 ± 1.45	207.15 ± 1.24	210.81 ± 1.37	219.44 ± 1.38	227.18 ± 1.58
**250 mg/kg**	205.13 ± 2.95	208.52 ± 2.56	212.35 ± 2.21	219.40 ± 1.53	227.09 ± 1.34
**1000 mg/kg**	204.18 ± 1.90	207.45 ± 1.63	211.07 ± 1.87	219.14 ± 1.22	225.16 ± 2.58
**2000 mg/kg**	204.92 ± 1.40	208.32 ± 2.51	211.57 ± 2.47	219.06 ± 2.48	226.56 ± 2.45

Values are expressed as mean ± SD. *n* = 5. No significant difference between the weights compared to the control group at 28 days

The hematological results obtained on male and female albino rats after the administration of three alternative doses of GlucoMedix® are shown in Tables 4 and 5. The only significant (*p* < 0.05) changes were increases in hematocrit (HCT), hemoglobin (HGB), and red blood cells (RBC) in male rats at 1000 and/or 2000 mg/kg, and an increase in hematocrit in female rats at 2000 mg/kg. These minor increases are not considered as detrimental. In both female and male rats there is no alteration in the values ​​of neutrophils (NEU), monocytes (MONO), eosinophils (EOS) and lymphocytes (LYM). The values ​​of white blood cells in female and male rats after the application of GlucoMedix® at doses of 250, 1000 and 2000 mg/kg demonstrated no significant differences compared to the control group (without treatment).

**Table 4:** Hematological value of male rats treated with GlucoMedix® at 28 days

Group/dose (mg/kg)	HCT (%)	HGB (g/dl)	WBC Differential Counting				RBC x10^**6**^cell/uL	WBC x10^**3**^cell/uL
			NEU	MON	EOS	LYM		
			(%)	(%)	(%)	(%)		
**250**	41.46 ± 0.80	13.90 ± 0.32	59.00 ± 2.92	4.00 ± 1.00	2.40 ± 0.55	32.80 ± 3.11	4.64 ± 0.38	7.16 ± 0.34
**1000**	43.44 ± 0.88 *	14.76 ± 0.52	59.60 ± 3.21	3.80 ± 0.84	1.60 ± 0.55	35.20 ± 2.86	4.96 ± 0.18*	6.98 ± 0.16
**2000**	44.54 ± 0.75 *	15.82 ± 0.61*	59.40 ± 2.88	4.20 ± 0.84	1.60 ± 0.89	35.00 ± 3.32	5.04 ± 0.21*	7.36 ± 0.29
**Control**	40.94 ± 0.60	13.84 ± 0.50	59.20 ± 2.77	3.40 ± 0.55	1.80 ± 0.45	35.60 ± 2.07	4.06 ± 0.26	6.94 ± 0.21

Values are expressed as mean ± SD. *n =* 5. Parameters include *HCT* Hematocrit, *HGB* Hemoglobin, *NEU* Neutrophils, *MON* Monocytes, *EOS* Eosinophils, *LYM* Lymphocytes, *RBC* Red blood cells, and *WBC* White blood cells. * *p* < 0.05 compared to control group

**Table 5:** Hematological value of female rats treated with GlucoMedix® at 28 days

Group/dose (mg/kg)	HCT (%)	HGB (g/dl)	WBC Differential Counting				RBC x10^**6**^cell/ul	WBC x10^**3**^cell/ul
			NEU	MONO	EOS	LYM		
			%	(%)	(%)	(%)		
**250**	41.06 ± 1.14	13.66 ± 0.44	60.2 ± 1.64	4.60 ± 1.14	2.80 ± 0.84	31.00 ± 3.08	4.46 ± 0.27	6.26 ± 0.23
**1000**	42.98 ± 3.61	13.70 ± 0.34	58.00 ± 2.55	4.80 ± 1.30	2.40 ± 0.55	34.80 ± 2.95	4.92 ± 0.24	6.10 ± 0.19
**2000**	43.24 ± 0.42 *	14.48 ± 0.53	60.20 ± 1.10	4.20 ± 1.30	2.60 ± 0.55	29.80 ± 4.60	4.70 ± 0.32	6.08 ± 0.24
**Control**	40.08 ± 0.80	13.32 ± 0.51	60.40 ± 1.14	4.40 ± 0.89	2.60 ± 0.55	31.60 ± 2.07	4.64 ± 0.32	6.08 ± 0.22

Values are expressed as mean ± SD. *n =* 5. Parameters include hematocrit (HCT), hemoglobin (HGB), neutrophils (NEU), monocytes (MON), eosinophils (EOS), lymphocytes (LYM), red blood cells (RBC), and white blood cells (WBC). * *p* < 0.05 compared to control group

Tables 6 and 7 show the results obtained from the clinical biochemistry on male and female albino rats after the administration of variable doses of GlucoMedix®. No effects were observed at the doses tested on the values ​​of glucose (GLU), cholesterol (T-Chol), and triglycerides (TG) compared to the control group. GlucoMedix® at the maximum dose of 2000 mg/kg does not alter the function of transaminases (AST and ALT), as well as the urea (BUN) and creatinine (Crea) values ​​compared to the control group (without treatment). The hormones TSH, T3 and T4 are not altered after 28 days in both sexes.

**Table 6:** Clinical chemistry of male rats treated with GlucoMedix® at 28 days

Group/dose (mg/kg)	GLU (mg/dL)	T-Chol (mg/dL)	TG (mg/dL)	AST(U/L)	ALT(U/L)	BUN (mg/dL)	Crea (mg/dL)	TSH (mIU/L)	T3(mIU/L)	T4(ug/dL)
**250**	92.2 + 2.59	97.2 + 1.64	70.0 + 1.58	31.4 + 0.89	34.8 + 0.84	37.6 + 2.07	0.84 + 0.17	2.98 + 0.32	2.88 + 0.15	1.14 + 0.15
**1000**	90.4 + 1.52	92.8 + 4.15	67.4 + 1.52	29.4 + 1.14	33.2 + 2.59	37.2 + 1.64	0.64 + 0.11	2.88 + 0.31	2.68 + 0.16	1.02 + 0.13
**2000**	87.2 + 2.59	90.8 + 3.56	64.6 + 2.70	27.6 + 2.97	32.8 + 1.64	34.6 + 2.61	0.66 + 0.15	2.88 + 0.43	2.34 + 0.17	0.94 + 0.11
**Control**	90.0 + 1.58	94.2 + 3.19	65.4 + 3.51	28.2 + 2.86	35.2 + 2.77	35.6 + 3.36	0.80 + 0.07	2.82 + 0.41	2.44 + 0.18	1.04 + 0.13

Values are expressed as mean ± SD. *n =* 5. Parameters included: glucose (GLU), total cholesterol (T-Chol), triglycerides (TG), aspartate aminotransferase (AST), alanine aminotransferase (ALT), blood urea nitrogen (BUN), creatinine (Crea), thyroid stimulating hormone (TSH), and thyroid hormones (T3, T4). No significant difference between the parameters compared to the control group

**Table 7:** Clinical chemistry of female rats treated with GlucoMedix® at 28 days

Group/dose (mg/kg)	GLU (mg/dL)	T-Chol (mg/dL)	TG (mg/dL)	AST (U/L)	ALT (U/L)	BUN (mg/dL)	Crea (mg/dL)	TSH (mIU/L)	T3 (mIU/L)	T4 (ug/dL)
**250**	90.6 + 3.36	94.0 + 1.58	68.0 + 2.35	30.8 + 1.30	33.2 + 0.84	39.4 + 1.67	0.74 + 0.15	2.90 + 0.33	2.68 + 0.08	1.00 + 0.19
**1000**	87.0 + 2.74	95.2 + 1.92	64.6 + 1.67	29.8 + 1.30	31.6 + 2.07	37.0 + 1.58	0.7 + 0.16	2.76 + 0.38	2.54 + 0.23	0.90 + 0.27
**2000**	85.8 + 3.19	89.2 + 1.64	64.0 + 1.58	25.0 + 2.55	31.2 + 1.30	32.6 + 2.19	0.72 + 0.18	2.96 + 0.44	2.28 + 0.24	0.84 + 0.18
**Control**	85.0 + 4.18	90.2 + 4.82	62.8 + 1.92	25.6 + 2.70	34.6 + 3.36	33.6 + 2.61	0.7 + 0.14	2.80 + 0.30	2.32 + 0.28	0.90 + 0.12

Values are expressed as mean ± SD. *n =* 5. Parameters included: glucose (GLU), total cholesterol (T-Chol), triglycerides (TG), aspartate aminotransferase (AST), alanine aminotransferase (ALT), blood urea nitrogen (BUN), creatinine (Crea), thyroid stimulating hormone (TSH), and thyroid hormones (T3, T4). No significant difference between the parameters compared to the control group

Necropsy revealed, no abnormal gross findings in either sex. No significant adverse changes in relative organ weights were observed for male and female rats. Histopathological analyzes show no observable damage to the evaluated organs: lungs, heart, stomach, intestine, testes, liver, kidney, and bladder. The group treated with GlucoMedix® at a maximum dose of 2000 mg/kg presented normal evaluated organs and vascular structures without significant histological alterations compared to the control group.

The maximum dose tested is 2 x higher than the maximum dose used in the three efficacy models (below). Thus, any pharmacologic efficacy observed in the three efficacy models (below) is not a coincidental undesirable consequence of a toxicologic effect. In other words, the mechanism(s) of action (MOA) for the three efficacy endpoints are not mediated via toxicity. The combined results of the acute toxicity up to 5000 mg/kg (above) and the subacute toxicity up to 2000 mg/kg indicate an acceptable safety profile for GlucoMedix®, and without any toxicologic properties.

### Anti-hyperglycemic activity

The GlucoMedix® treatments produced an anti-hyperglycemic activity in the alloxan-induced rat model, at doses of 250, 500, and 1000 mg/kg of body weight, and in a dosage-dependent manner, and the values were significant at 28 days (*p* < 0.05 or < 0.001). The data are shown in Table 8. Glibenclamide (10 mg/kg) was the positive control.

**Table 8:** Effect of GlucoMedix® on glucose concentrations in alloxan-induced hyperglycemic rats

Group/dose (mg/kg)	Glucose (mg/dl)					
	BASAL	0 days	7 days	14 days	21 days	28 days
Control (uninduced)	89.0 ± 2.90	110.2 ± 5.53	106.6 ± 8.50	109.2 ± 8.63	112.6 ± 6.95	114.8 ± 6.05
Negative Control (induced)	90.2 ± 3.19	392.8 ± 18.62**	419.8 ± 15.99**	447.6 ± 15.85**	465.4 ± 9.81**	482.8 ± 2.59 **
Glibenclamide 10	88.6 ± 3.36	347.2 ± 28.80**	233.8 ± 14.72*	166.8 ± 14.17*&&	126.6 ± 15.19 &&	94.2 ± 5.45 *&&
GlucoMedix 250	90.4 ± 1.85	363.4 ± 28.75**	340.8 ± 26.90**&	318.4 ± 18.03**&	292.6 ± 38.49*&	248.8 ± 26.68 **&
GlucoMedix 500	95.8 ± 3.43	413.0 ± 14.75**	390.6 ± 14.11**	333.8 ± 11.50**&	257.4 ± 15.59*&	164.6 ± 9.77 *&&
GlucoMedix 1000	90.6 ± 3.65	349.8 ± 25.04**	281.4 ± 14.96*&	228.0 ± 8.03*&	169.4 ± 10.29*&	105.2 ± 4.15 *&&

Values are expressed as mean ± SD. *n =* 7. * *p* < 0.05, ** *p* < 0.001 compared to control group; & *p* < 0.05, && *p* < 0.001 compared to negative control

At 28 days the uninduced (basal) level of circulating glucose was 114.8 ± 6.05 mg/dl and the induced (hyperglycemic) level was 482.8 ± 2.59 mg/dl. Glibenclamide (10 mg/kg) reduced glucose to 94.2 ± 5.45 mg/dl and GlucoMedix® (1000 mg/kg) reduced it to 105.2 ± 4.15 mg/dl. Thus, both the pharmaceutical control and highest dose of GlucoMedix® completely abrogated the induced (hyperglycemic) parameter.

### Anti-hyperlipidemic activity

**Fig. 3:**
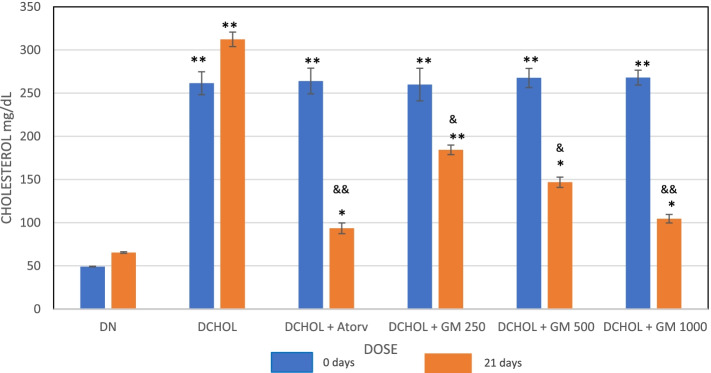
Effect of GlucoMedix® on cholesterol levels at 21 days of treatment in hypercholesterolemic rats. Values are expressed as mean ± SD. *n* = 7. DN = Diet normal control; DCHOL = Cholesterol induced control; DCHOL + Atorv = Atorvastatin (20 mg/kg); DCHOL + GM 250, DCHOL + GM 500 and DCHOL + GM 1000 = GlucoMedix® at 250, 500 and 1000 mg/kg respectively. * *p* < 0.05, ** *p* < 0.001 compared to diet control group; & *p* < 0.05, && *p* < 0.001 compared to cholesterol induced control

**Fig. 4:**
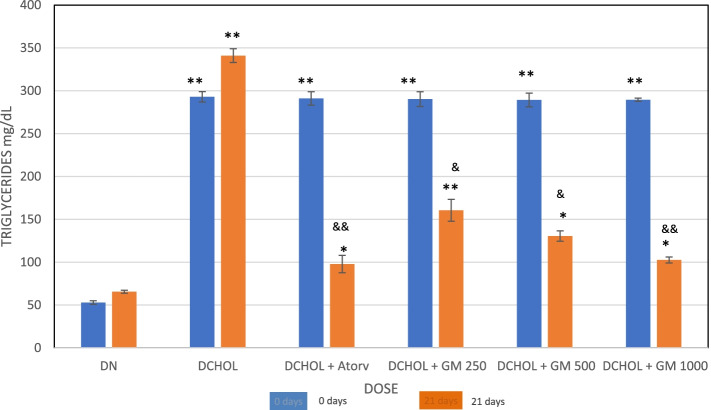
Effect of GlucoMedix® on triglyceride levels at 21 days of treatment in hypercholesterolemic rats. Values are expressed as mean ± SD. *n =* 7. DN = Diet normal control; DCHOL = Cholesterol induced control; DCHOL + Atorv = Atorvastatin (20 mg/kg); DCHOL + GM 250, DCHOL + GM 500 and DCHOL + GM 1000 = Glucomedix at 250, 500 and 1000 mg/kg respectively. * *p* < 0.05, ** *p* < 0.001 compared to diet control group; & *p* < 0.05, && *p* < 0.001 compared to negative control

GlucoMedix® treatment produced a decrease in the cholesterol and triglyceride levels in cholesterol-induced hyperlipidemic rats at Day 21 at doses of 250, 500, and 1000 mg/kg, and in a dosage-dependent manner, and the values were significant at 21 days (*p* < 0.05 or < 0.001). The data are shown in Figs. 3 and 4 and Tables 9 and 10. The positive control was atorvastatin, an HMG-CoA reductase inhibitor.

**Table 9:** Effect of GlucoMedix® on cholesterol levels of normal and hyperlipidemic rats

Group/dose (mg/kg)	Basal	Post-induction	21 days
Control (uninduced)	48.05 ± 0.64	49.12 ± 0.41	65.37 ± 0.87
Negative Control (induced)	51.94 ± 0.53	261.48 ± 13.33	312.31 ± 8.36 **
Atorvastatin 20	50.86 ± 1.13	264.02 ± 14.94	93.48 ± 6.17 * &&
Glucomedix 250	53.63 ± 0.72	259.89 ± 18.89	184.28 ± 5.58 ** &
Glucomedix 500	52.29 ± 1.04	267.61 ± 11.02	146.83 ± 5.96 * &
Glucomedix 1000	49.91 ± 0.98	268.01 ± 8.61	104.56 ± 4.87 * &&

Values are expressed in mg/dl as mean ± SD. *n* = 7. * *p* < 0.05, ** *p* < 0.001 compared to control group; & *p* < 0.05, && *p* < 0.001 compared to negative control

**Table 10:** Effect of GlucoMedix® on triglyceride levels of normal and hyperlipidemic rats

Group/dose (mg/kg)	Basal	Day 1	Day 21
Control (uninduced)	53.05 ± 1.12	52.89 ± 2.12	65.56 ± 1.76
Negative Control (induced)	56.12 ± 0.87	292.92 ± 6,04	340.98 ± 8.04 **
Atorvastatin 20	58.73 ± 1.10	291.08 ± 7.82	97.82 ± 10.13 *&&
Glucomedix 250	61.32 ± 0.94	290.24 ± 8,61	160.55 ± 12,76 **&
Glucomedix 500	60.53 ± 1.01	289.28 ± 8,01	130.49 ± 6.02 * &
Glucomedix 1000	59.11 ± 0.78	289.55 ± 1.86	102.55 ± 3.47 * &&

Values are expressed in mg/dl as mean ± SD. *n =* 7. * *p* < 0.05, ** *p* < 0.001 compared to control group; & *p* < 0.05, && *p* < 0.001 compared to negative control

In Table 9 the uninduced (baseline) level of circulating cholesterol at 21 days was 65.37 ± 0.87 mg/dl and the induced (hyperlipidemic) level was 312.31 ± 8.36 mg/dl. GlucoMedix® (1000 mg/kg) reduced cholesterol to only 104.56 ± 4.87 mg/dl, a concentration slightly less effective than the pharmaceutical control Atorvastatin (20 mg/kg) at 93.48 ± 6.17 mg/dl.

In Table 10 the uninduced (baseline) level of triglycerides at 21 days was 65.56 ± 1.76 mg/dl and the induced (hyperlipidemic) level was 340.98 ± 8.04 mg/dl. GlucoMedix® (1000 mg/kg) reduced triglycerides to 102.55 ± 3.47 mg/dl, a concentration close to that achieved by atorvastatin (20 mg/kg) at 97.82 ± 10.13 mg/dl.

### Antihypertensive activity

**Fig. 5:**
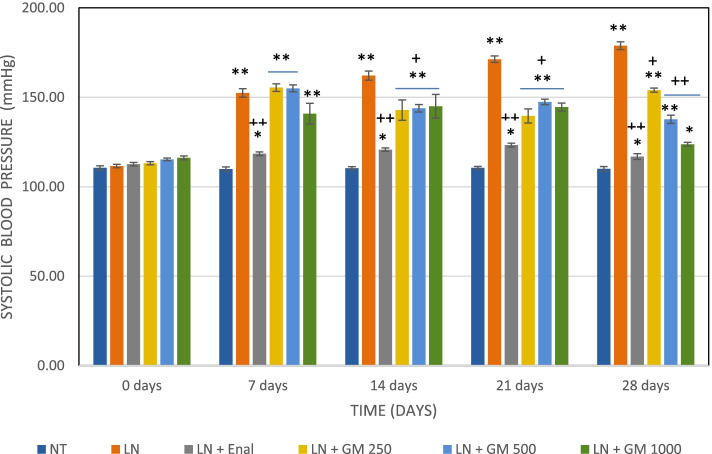
Effects of GlucoMedix® on systolic blood pressure in hypertensive rats induced by L-NAME. Values are expressed as mean ± SD. *n =* 7; NT = normotensive control; LN = L-NAME induced control; LN + Enal = Enalapril; LN + GM 250, LN + GM 500 and LN + GM 1000 = GlucoMedix at 250, 500 and 1000 mg/kg respectively. * *p* < 0.05, ** *p* < 0.001 compared to normotensive control group; +*p* < 0.05, ++*p* < 0.001 compared to L-NAME (LN) induced control group

**Fig. 6:**
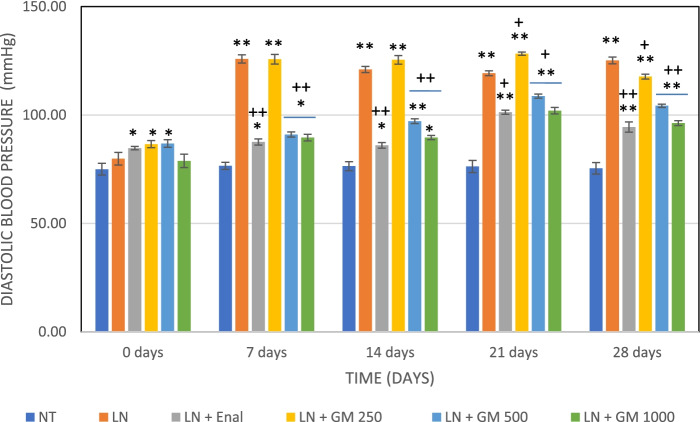
Effects of GlucoMedix® on diastolic blood pressure in hypertensive rats induced by L-NAME. Values are expressed as mean ± SD. *n =* 7; NT = normotensive control; LN = L-NAME induced control; LN + Enal = Enalapril; LN + GM 250, LN + GM 500 and LN + GM 1000 = GlucoMedix at 250, 500 and 1000 mg/kg respectively. * *p* < 0.05, ** *p* < 0.001 compared to normotensive control group; +*p* < 0.05, ++*p* < 0.001 compared to L-NAME (LN) induced control group

**Fig. 7:**
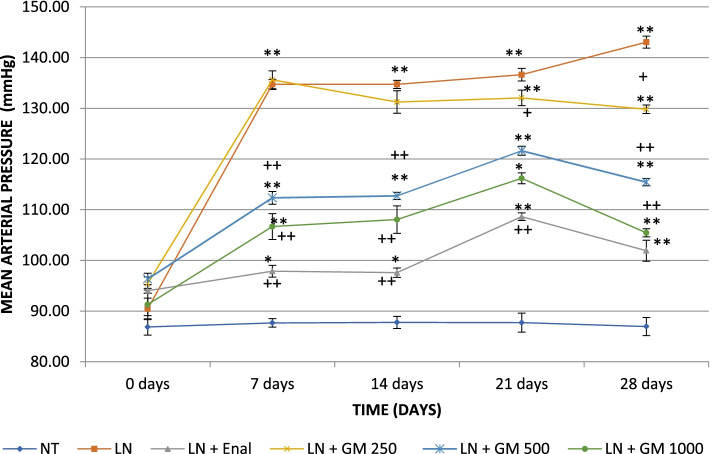
Effects of GlucoMedix® on mean blood pressure in hypertensive rats induced by L-NAME. Values are expressed as mean ± SD. *n =* 7; NT = normotensive control; LN = L-NAME induced control; LN + Enal = Enalapril; LN + GM 250, LN + GM 500 and LN + GM 1000 = GlucoMedix at 250, 500 and 1000 mg/kg respectively. * *p* < 0.05, ** *p* < 0.001 compared to normotensive control group; +*p* < 0.05, ++*p* < 0.001 compared to L-NAME (LN) induced control group

Systolic and diastolic blood pressures increased significantly in all treatment groups compared to the baseline (day 0) following 7 days of L-NAME (non-selective NOS inhibitor) treatment. The untreated L-NAME group at 28 days showed a rise in the value of the systolic pressure to 178.9 ± 2.19 mmHg and for the diastolic pressure to 125.1 ± 1.57 mmHg. GlucoMedix® showed dose-dependent antihypertensive activity as can be seen in the Figs. 5, 6, 7 and Tables 11, 12, 13, for systolic, diastolic, and mean blood pressures.

**Table 11:** Effects of Glucomedix® on systolic blood pressure in hypertensive rats induced by L-NAME

Group/dose (mg/kg)	Time (days)				
	0 days	7 days	14 days	21 days	28 days
NT	110.6 ± 1.13	109.9 ± 1.21	110.4 ± 0.79	110.6 ± 0.79	110.0 ± 1.29
LN	111.6 ± 0.98	152.4 ± 2.37**	162.1 ± 2.54**	171.3 ± 1.80 **	178.9 ± 2.19 **
LN + Enal 25	112.6 ± 0.98	118.4 ± 0.98*++	120.7 ± 0.95*++	123.3 ± 1.11 *++	116.9 ± 1.57 *++
LN + GM 250	113.1 ± 0.90	155.4 ± 2.15**	142.9 ± 5.70**+	139.6 ± 3.95**+	154.0 ± 1.15 **+
LN + GM 500	115.3 ± 0.76	155.0 ± 1.91**	143.9 ± 2.12**+	147.4 ± 1.51**+	137.7 ± 2.29 **++
LN + GM 1000	116.1 ± 1.07	140.9 ± 5.79**	145.0 ± 6.58**+	144.6 ± 2.23**+	123.7 ± 1.11 *++

Values are expressed as mean ± SD. *n =* 7; NT = normotensive control; LN = L-NAME induced control; LN + Enal = Enalapril; LN + GM 250, LN + GM 500 and LN + GM 1000 = GlucoMedix at 250, 500 and 1000 mg/kg respectively. * *p* < 0.05, ** *p* < 0.001 compared to normotensive control group; +*p* < 0.05, ++*p* < 0.001 compared to L-NAME (LN) induced control group

**Table 12:** Effects of Glucomedix® on diastolic blood pressure in hypertensive rats induced by L-NAME

Group/dose (mg/kg)	Time (days)				
	0 days	7 days	14 days	21 days	28 days
NT	75.0 ± 2.71	76.6 ± 1.62	76.4 ± 2.07	76.3 ± 2.81	75.4 ± 2.64
LN	79.9 ± 2.91	125.9 ± 1.86**	121.0 ± 1.41 **	119.3 ± 1.11 **	125.1 ± 1.57 **
LN + Enal 25	84.7 ± 0.76 *	87.6 ± 1.40 *++	86.0 ± 1.29 *++	101.3 ± 0.95 **+	94.4 ± 2.37 **++
LN + GM 250	86.6 ± 1.62*	125.7 ± 2.21**	125.4 ± 1.99 **	128.3 ± 0.76**+	117.7 ± 1.11 **+
LN + GM 500	86.9 ± 1.77*	91.0 ± 1.15*++	97.1 ± 1.07**++	108.7 ± 0.95**+	104.3 ± 0.76**++
LN + GM 1000	78.9 ± 3.08	89.6 ± 1.51*++	87.6 ± 0.98 *++	102.0 ± 1.53**+	96.3 ± 1.11 **++

Values are expressed as mean ± SD. *n =* 7; NT = normotensive control; LN = L-NAME induced control; LN + Enal = Enalapril; LN + GM 250, LN + GM 500 and LN + GM 1000 = GlucoMedix at 250, 500 and 1000 mg/kg respectively. * *p* < 0.05, ** *p* < 0.001 compared to normotensive control group; +*p* < 0.05, ++*p* < 0.001 compared to L-NAME (LN) induced control group

**Table 13:** Effects of Glucomedix® on mean blood pressure in hypertensive rats induced by L-NAME

Group/dose (mg/kg)	Time (days)				
	0 days	7 days	14 days	21 days	28 days
NT	86.9 ± 1.59	87.7 ± 0.84	87.8 ± 1.20	87.7 ± 1.87	87.0 ± 1.79
LN	90.4 ± 2.12	134.7 ± 1.01**	134.7 ± 0.80**	136.6 ± 1.24**	143.1 ± 1.19 **
LN + Enal 25	94.0 ± 0.47	97.9 ± 1.17*	97.6 ± 0.92*++	108.6 ± 0.76*++	101.9 ± 2.07 *++
LN + GM 250	95.4 ± 1.29	135.6 ± 1.77**	131.2 ± 2.23**	132.1 ± 1.53**+	129.8 ± 0.84 **+
LN + GM 500	96.3 ± 1.12	112.3 ± 1.26*+	112.7 ± 0.71*+	121.6 ± 0.89*+	115.4 ± 0.71 *+
LN + GM 1000	91.3 ± 2.19	106.7 ± 2.55*++	108.1 ± 2.70*++	116.2 ± 1.07*++	105.4 ± 0.81 *++

Values are expressed as mean ± SD. *n =* 7; NT = normotensive control; LN = L-NAME induced control; LN + Enal = Enalapril; LN + GM 250, LN + GM 500 and LN + GM 1000 = GlucoMedix at 250, 500 and 1000 mg/kg respectively. * *p* < 0.05, ** *p* < 0.001 compared to normotensive control group; +*p* < 0.05, ++*p* < 0.001 compared to L-NAME (LN) induced control group

The groups of rats treated with the pharmaceutical control Enalapril (25 mg/kg) and GlucoMedix® (250, 500, and 1000 mg/kg) showed significant (*p* < 0.05 or < 0.001) decreases at 28 days in the values of the systolic, diastolic, and (calculated) mean blood pressures. At 28 days the pharmaceutical control Enalapril vs. GlucoMedix® (1000 mg/kg) blood pressures were 116.9 ± 1.57 mmHg systolic / 94.4 ± 2.37 mmHg diastolic vs. 123.7 ± 1.11 mmHg / 96.3 ± 1.11 mmHg, respectively. Thus, the highest dose of GlucoMedix® was close to the efficacy of the positive control ACE inhibitor.

### Pharmacologic dose responses

**Fig. 8:**
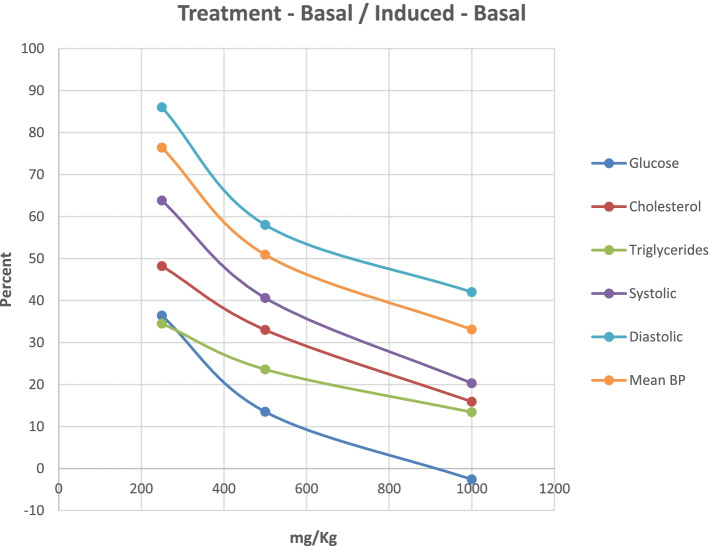
Dose response for oral GlucoMedix® in rat models. GlucoMedix® (250, 500, 1000 mg/kg) treatment effects, expressed as the percentage of the chemically induced maximum minus the uninduced baseline; glucose at 28 days; cholesterol and triglycerides at 21 days; systolic, diastolic, and mean BP at 28 days

In Fig. 8, the dose responses of the various rat efficacy models are summarized. The treatment effects of GlucoMedix® are expressed as the percentage of the chemically induced maximum levels minus the uninduced baseline levels in each animal model. Herein, 100% represents no inhibition of the induced parameter, whereas 0% is total inhibition of the induced parameter (i.e., reduction to baseline). The glucose result is from 28 days of treatment; the cholesterol and triglycerides are from 21 days; and the blood pressures are from 28 days. It is unknown whether any further reductions in cholesterol and/or triglycerides would be achieved by an additional week of treatment (i.e., from 21 to 28 days).

Dose response curves are evident for each of the animal models tested, with the anti-hyperglycemic effect of GlucoMedix® being the most potent when comparing the three independent animal models. Note within the prior three sections (above) the relative effectiveness of the highest dose of GlucoMedix® vs. the three pharmaceutical positive controls - Enalapril, Atorvastatin, and Glibenclamide. Even at the lowest oral dose of 250 mg/kg of the *Uncaria* plus *Stevia* extract, there is evidence of reductions in blood pressure, lipids, and glucose.

If Inhibitory Concentration 50% (IC_50_) values are applied to GlucoMedix®, then the IC_50_ values for glucose, cholesterol, and triglycerides in these selected rat models are below 250 mg/kg, and for mean blood pressure it is approximately 500 mg/kg. Thus, to achieve a minimum of half-maximal inhibition in a genetic or diet-induced rat model of metabolic syndrome per se (i.e., manifesting multiple comorbid conditions) and for all of the endpoints assessed herein in individual rat models, then the recommended dose is 500 mg/kg. If total pharmacologic blockade is desired (and in this duration of treatment), then the Inhibitory Concentration 100% (IC_100_) values in the selected rat models herein would be approximately 1000 mg/kg for glucose, and greater than 1000 mg/kg for cholesterol, triglycerides, and mean BP. Also, note that 1000 mg/kg dosing is comparable to the clinical effect of the pharmaceutical positive controls. Furthermore, these IC_50_ values provide guidance toward allometrically-scaled starting oral dosing in humans (see Discussion below).

## Discussion

GlucoMedix® does not produce acute toxic effects in rats; the LD50 being greater than 5.0 g/Kg. Also, in 28-day subacute toxicity studies we did not observe mortality or signs of toxicity, and no significant weight loss was registered. Therefore, the NOAEL for the subacute toxicity study was 2000 mg/kg. According to the dosage levels evaluated in the subacute and acute toxicity studies, the LOAEL (Lowest Observed Adverse Effect Level) was not found. The only statistically significant effects in the 28-day oral treatments were minor increases in hematocrit, hemoglobin, and red blood cells in males and hematocrit in females. Thus, GlucoMedix® could be considered with a wide margin of safety for oral use in humans.

Regarding efficacy in three animal models, GlucoMedix® reduced the systolic and diastolic arterial pressure in hypertensive animals, which was induced by L-NAME, as evidenced with a 28-day treatment. In hyperglycemic and hyperlipidemic animals treated with GlucoMedix® substantial and statistically significant beneficial effects were observed. All three rodent efficacy models manifested potent and dose dependent effects at 250 - 1000 mg/kg (extract wet weight), thus demonstrating pharmacologic benefits without any coincident adverse toxicities. The highest dose (1000 mg/kg) was comparable to the pharmaceutical positive controls.

Various pharmacologic mechanisms of action (MOAs) of GlucoMedix® are plausible for reducing glucose, lipids, triglycerides, and blood pressure in the rat animal models.

*Stevia* and steviol glycosides might down-regulate the levels of glucose and lipids in blood, as well as arterial hypertension. *Stevia* phytochemicals or steviol glycosides were known in human clinical trials to affect type 2 diabetes [11, 15]. There is evidence of a possible benefit regarding hypertension in humans [5]. However, another study of only 7 patients per group yielded a negative result for hypertension [4], although statistically significant reductions in cholesterol, LDL, and glucose were observed.

The *Stevia*-derived ingredients were also effective in rat models in alloxan-induced hyperglycemia [13, 14, 17], streptozotocin-induced hyperglycemia [18], and cholesterol-induced hyperlipidemia [12]. Another rat study showed that stevioside and powdered *Stevia* leaves in high-carbohydrate and high-fat diets caused a significant reduction in blood glucose level after 4 weeks of treatment [40]. Our studies in three rat efficacy models are consistent with these prior findings, presuming that the steviol glycosides are contributing to the overall efficacy of GlucoMedix®.

*Uncaria* extracts have been found to reduce glucose levels in mice and rat animal models [32, 33]. A hydro-alcoholic extract of *Uncaria* containing POAs (29.1 mg/g) in a streptozotocin-induced mouse model, showed a reduction in glycemic levels [33]. Likewise, rats treated with 75 and 150 mg/kg of *Uncaria tomentosa* dry extract showed a reduction in blood glucose [32]. One possible MOA for this glucose down-regulation is explained by alpha-glucosidase and alpha-amylase inhibitory activities within *Uncaria* extracts [41, 42]. These enzymes catalyze the hydrolysis of complex polysaccharides, such as dietary starch and endogenous glycogen. This enzymatic antagonism of biodegradation of polysaccharide precursors might reduce blood glucose, and thus possibly contribute to the overall glycemic regulatory efficacy of GlucoMedix®.

Steviol glycosides and/or *Uncaria* phytochemicals might be affecting the endocrine and/or neuro-endocrine system, and in particular the hypothalamic-pituitary-adrenal (HPA) axis. Cortisol levels might be a possible mediator under the influence of these bioactive compounds. Cortisol is known to play a key role in glucose utilization. Patients with metabolic syndrome exhibit elevated HPA axis properties leading to hypercortisolism [43, 44]. Future studies of GlucoMedix® could assess levels of cortisol and insulin.

Another possible MOA is that the *Uncaria* POAs are affecting the immune system [33, 36, 45, 46]. However, it should be noted that the subacute toxicology study at doses as high as 2000 mg/kg for four weeks did not reveal any significant alterations in white blood cell numbers or ratios. If the MOA is immunomodulatory, it is not being achieved by altering the number of white blood cells.

Regardless of the MOA, one significant factor to consider is that the three rodent efficacy models involved experimental induction agents (i.e., alloxan, L-NAME, and cholesterol) that result in parameters exceeding normal physiologic levels, whereas the acute and subacute toxicology models were not dependent on any induction events. In other words, the toxicity model was performed in a natural physiologic state. The 28-day toxicity studies further underscore that any efficacy benefit in hyper-normal physiological states (e.g., induced states or disease states) is not expected to result in any adverse outcome extending below baseline parameters in normal laboratory animals (or humans).

A beneficial aspect of these animal model efficacy and toxicity studies run in parallel is the establishment of a favorable therapeutic index. In other words, GlucoMedix® achieved the desired efficacy endpoints without any observable toxicity at or above the effective dose(s) and at coincident time points (i.e., at 3-4 weeks).

Toxicologic studies in rodents have demonstrated the safety of extracts and isolated compounds of *Uncaria tomentosa* and *Stevia rebaudiana* [13, 25, 47]. Our study shows that GlucoMedix® has an LD50 in rats greater than 5000 mg/kg of body weight, and well-being parameters such as sleep, behavior pattern, motor activity, skin, coat, and appetite were normal. No weight loss was observed after two weeks of observation.

Metabolic syndrome is often associated with type 2 diabetes, but it can exist in patients lacking this comorbidity. In the US a diagnosis typically involves any three of five comorbidities, as per the NCEP-ATP III criteria. Although type 2 diabetes is common, it is not the essential factor driving the pathophysiology of metabolic syndrome in all patients.

Although some articles assert that alloxan induction is an experimental model for type 2 diabetes [14, 17], it should be noted that the alloxan-induced and glibenclamide-controlled rat model is more closely related to type 1 diabetes (insulin insufficiency), rather than type 2 diabetes (insulin resistance). This suggests that GlucoMedix® might be stimulating production of insulin from the remaining pancreatic beta cells following toxic damage to the tissue by alloxan. Thus, this animal model does not provide a precise correlate for type 2 diabetes within metabolic syndrome. Beyond the scope of the present experiment, two relevant rodent models for consideration for future confirmatory studies are C57BL6J male mice on high fat diet and Zucker Diabetic Fatty (ZDF) rats [48–50].

*Stevia* extract has long been used for the treatment of diabetes in South America [51]. Furthermore, stevioside is a potent sweetener with no calories. Thus, *Stevia*-derived products can achieve reductions in blood glucose in humans by two means: (a) as a substitute for dietary sugars, thus reducing ingested sugars; and (b) as a pharmacologic active ingredient affecting glucose homeostasis.

The GlucoMedix® extract of *Uncaria* and *Stevia* shows anti-hyperglycemic activity in alloxan-induced rats treated at doses of 250 - 1000 mg/Kg of body weight. GlucoMedix® might regulate the level of glucose by increasing insulin secretion and/or by a better utilization of glucose by peripheral tissues and muscles in diabetic rats.

One of the most common complications of diabetes mellitus is cardiovascular disease. Other studies have suggested that *Uncaria*, *Stevia*, and their metabolites promote cardiovascular health and reduce hypertension. Our results with GlucoMedix® show a decrease in cholesterol and triglyceride levels in hyperlipidemic rats at 21 days and a decrease in blood pressure induced by L-NAME in hypertensive rats at 28 days of treatment with doses of 250 - 1000 mg/Kg.

The 1000 mg/kg daily dose (wet weight) is equivalent to administering 81.8 mg of steviol glycosides, 16.98 μg of isopteropodine, and 4.71 μg of pteropodine per kg of body weight in rats. This maximum tested dose of GlucoMedix® displayed the same or similar potency to the three pharmaceutical positive controls. However, comparison of dosing to other published rodent models treated with other extracts is somewhat problematic. For example, Ahmad and coworkers demonstrated anti-hyperlipidemic effects in cholesterol-induced rats using 200 - 500 ppm of *Stevia* extract; presumably this represents 200 - 500 mg/kg dry weight of *Stevia* powder [12]. Thus, they tested 200 - 500 mg/kg vs. 29.2 - 116.7 mg/kg of *Stevia* powder within GlucoMedix® in the present study. As another example, Kujur and coworkers demonstrated anti-hyperglycemic effects in alloxan-induced rats using 50 - 100 mg/kg (wet weight) of *Stevia* extracts at 28 days; the dry weights are unknown [13].

If the 250, 500, and 1000 mg/Kg daily doses in rats (ca. 0.24 Kg) are extrapolated via allometric dosage conversion for oral administration in humans (65 Kg and 0.75 exponent), the corresponding allometric daily doses of GlucoMedix® required in an adult for “similar” pharmacologic effects would be 4, 8, and 16 g (wet weight).

Given that the extract mixture contains ca. 2.56 mg/100 ml of POAs, then these human allometric doses would contain only 0.10, 0.20, and 0.41 mg of POAs. Note that few active pharmaceutical ingredients (APIs) in the pharmacopeia are effective in the sub-milligram level in human adults. However, predicate examples do exist; an example is the phytochemical scopolamine that is effective at 0.1 - 0.5 mg in humans [52, 53]. If the POAs are contributing to the efficacy endpoints, then they would by necessity be highly potent phytochemicals.

Allometric dosage conversion presumes similarities between the two species regarding pathophysiology, pharmacokinetics, and pharmacodynamics. A suggested human starting oral daily dose of GlucoMedix® that might be effective within 4 weeks at treating metabolic syndrome or its comorbidities is 4 g. Given that the IC_50_ values for glucose and lipids (cholesterol and triglycerides) were below 250 mg/kg in rats, then it is reasonable to speculate that adult doses lower than 4 g (and/or with longer duration of treatment) might also be effective in humans.

A physician-sponsored retrospective case series study has been reported of six humans afflicted by type 2 diabetes, which were treated with GlucoMedix® at daily doses of 4 or 6 g [54]. The patients experienced reductions in hyperglycemia, and several of them coincidentally reduced or ceased treatments with prescription drugs or insulin. Thus, the suggested minimum allometric dose (4 g) based upon the rat efficacy model for hyperglycemia coincides with the minimum dosage used within the type 2 diabetes case series.

Furthermore, based upon the relative potencies in rats across the three indications summarized within Fig. 8, the most potent effect was observed for hyperglycemia (IC_50_ < 250 mg/kg), followed by hyperlipidemia (IC_50_ < 250 mg/kg), and finally mean blood pressure (IC_50_ ~ 500 mg/kg). If this correlation also applies in humans, it suggests that treatment of hypertension might require higher daily dosing with GlucoMedix® than is required for glucose regulation.

## Conclusions

Limitations of this work should be noted: (a) The pharmacologic effects might be due to *Stevia* alone, *Uncaria* alone, or the combination thereof; (b) The efficacy studies were based upon established chemical induction models, rather than genetic disease models that are predisposed to diabetes, hyperlipidemia, and hypertension. Future studies could also focus on alternative rodent models for hyperglycemia (and obesity) that mirror type 2 diabetes, such as C57BL6J male mice on high fat diet or Zucker Diabetic Fatty (ZDF) rats; and (c) The beneficial pharmacologic effects and lack of toxicity were assessed for up to 21 days (anti-hyperlipidemia) or 28 days (anti-hyperglycemia, anti-hypertension, and subacute toxicity).

A safe and effective natural product, such as GlucoMedix®, that can address multiple comorbidities of metabolic syndrome would be a welcome addition to the pharmacopeia and marketplace. We are unaware of any single US FDA-approved drug that can address all three conditions, although inexpensive monotherapies are available for treating hypertension (e.g., ACE inhibitors and beta blockers), hyperlipidemia (e.g., statins), and type II diabetes (e.g., metformin). A physician-sponsored case series of six type 2 diabetic patients suggests that this natural product at 1 - 1.5 x of the suggested starting allometrically-scaled dose can address at least one of the three indications, namely hyperglycemia [54].

## Data Availability

All data generated or analyzed during the study are included in this published article.
