# Correction: GlucoMedix®, an extract of Stevia rebaudiana and Uncaria tomentosa, reduces hyperglycemia, hyperlipidemia, and hypertension in rat models without toxicity: a treatment for metabolic syndrome

**DOI:** 10.1186/s12906-022-03708-9

**Published:** 2022-08-27

**Authors:** León F. Villegas Vílchez, Julio Hidalgo Ascencios, Thomas P. Dooley

**Affiliations:** 1 Department of Cellular and Molecular Sciences, Section of Pharmaceutical Sciences, Faculty of Sciences and Philosophy, Universidad Peruana Cayetano Heredia, Lima, Peru; 2 Quality Control Service, Research and Development Laboratories, Universidad Peruana Cayetano Heredia, Lima, Peru; 3 Research and Development, LivFul Inc, Cheshire, UK

**Correction: BMC Complement Med Ther 22, 62 (2022)**

**https://doi.org/10.1186/s12906-022-03538-9**

Following publication of the original article [1], the authors reported an error in Table 8 and Fig. 8.

The Table 8 reported for the glucose levels (Alloxan-induced) of GlucoMedix® treatments was incorrect. However, the correct data set reveals that the results are similar to those reported. The percentage of the chemically induced maximum level minus the uninduced level at 28 days (as displayed in Fig. 8) is 62.0 % at 250 mg/kg (vs 36.4 % as reported), 16.3 % at 500 mg/kg (vs 13.5 % as reported), and 0.8 % at 1,000 mg/kg (vs -2.6 % as reported), and the Glibenclamide 10 mg/kg positive control should be -1.2 % (vs -5.6 % as reported). Thus, the IC_50_ value for glucose levels with GlucoMedix® is between 250 and 500 mg/kg (vs < 250 mg/kg as reported). Furthermore, the number of animals per group for the glucose and lipid profile experiments was five (vs seven as reported).

**Table 8:** Effect of GlucoMedix® on glucose concentrations in alloxan-induced hyperglycemic rats

	Glucose (mg/dl)					
Group/dose (mg/kg)	BASAL	0days	7days	14days	21days	28days
**Control (uninduced)**	90.3 ± 1.49	92.0 ± 1.63 &&	93.3 ± 2.36 &&	94.3 ± 2.43 &&	96.2 ± 2.41 &&	98.5 ± 2.36 &&
**Negative Control (induced)**	92.7 ± 4.50	495.7 ± 20.62 **	528.2 ± 20.42 **	545.7 ± 15.91**	566.3 ± 12.34 **	579.0 ± 11.87 **
**Glibenclamide 10**	90.3 ± 2.34	500.0 ± 17.03 **	287.8 ± 18.76 *&	137.5 ± 8.78 *&&	107.3 ± 4.55 &&	92.7 ± 1.97 &&
**GlucoMedix 250**	90.2 ± 3.89	509.7 ± 13.83 **	482.5 ± 10.90 **&&	448.7 ± 12.49 **&	418.8 ± 16.10 **&	396.5 ± 13.38 **&
**GlucoMedix 500**	94.3 ± 3.77	512.5 ± 14.63 **	324.2 ± 17.06 **&	268.2 ± 15.83 *&	207.2 ± 2.54 *&&	177.0 ± 6.71*&&
**GlucoMedix 1000**	93.3 ± 3.33	501.5 ± 9.54 **	305.0 ± 8.00 **&	216.3 ± 4.93 *&&	146.2 ± 13.47 *&&	102.2 ± 4.02 &&

Values are expressed as mean ± SD. *n*= 5. Statistical significance was * *p*<0.05, ** *p*<0.001 compared to control group (uninduced); & *p*<0.05, && *p*<0.001 compared to negative control group (induced)

**Fig. 8:**
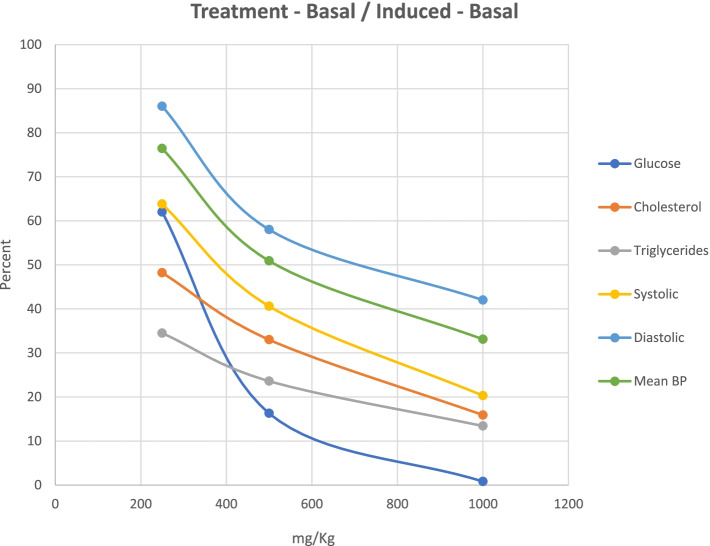
Dose response for oral GlucoMedix® in rat models. GlucoMedix® (250, 500, 1000 mg/kg) treatment effects, expressed as the percentage of the chemically induced maximum minus the uninduced baseline; glucose at 28 days; cholesterol and triglycerides at 21 days; systolic, diastolic, and mean BP at 28 days

The original article [1] has been updated.
